# New screening approach for Alzheimer’s disease risk assessment from urine lipid peroxidation compounds

**DOI:** 10.1038/s41598-019-50837-2

**Published:** 2019-10-02

**Authors:** Carmen Peña-Bautista, Claire Vigor, Jean-Marie Galano, Camille Oger, Thierry Durand, Inés Ferrer, Ana Cuevas, Rogelio López-Cuevas, Miguel Baquero, Marina López-Nogueroles, Máximo Vento, David Hervás-Marín, Ana García-Blanco, Consuelo Cháfer-Pericás

**Affiliations:** 1 Neonatal Research Unit, Health Research Institute La Fe, Valencia, Spain; 2 Institut des Biomolécules Max Mousseron, IBMM, University of Montpellier, CNRS ENSCM, Montpellier, France; 3 Neurology Unit, University and Polytechnic Hospital La Fe, Valencia, Spain; 4 Analytical Unit Platform, Health Research Institute La Fe, Valencia, Spain; 5 Biostatistical Unit, Health Research Institute La Fe, Valencia, Spain

**Keywords:** Diagnostic markers, Alzheimer's disease

## Abstract

Alzheimer Disease (AD) standard biological diagnosis is based on expensive or invasive procedures. Recent research has focused on some molecular mechanisms involved since early AD stages, such as lipid peroxidation. Therefore, a non-invasive screening approach based on new lipid peroxidation compounds determination would be very useful. Well-defined early AD patients and healthy participants were recruited. Lipid peroxidation compounds were determined in urine using a validated analytical method based on liquid chromatography coupled to tandem mass spectrometry. Statistical studies consisted of the evaluation of two different linear (Elastic Net) and non-linear (Random Forest) regression models to discriminate between groups of participants. The regression models fitted to the data from some lipid peroxidation biomarkers (isoprostanes, neuroprostanes, prostaglandines, dihomo-isoprostanes) in urine as potential predictors of early AD. These prediction models achieved fair validated area under the receiver operating characteristics (AUC-ROCs > 0.68) and their results corroborated each other since they are based on different analytical principles. A satisfactory early screening approach, using two complementary regression models, has been obtained from urine levels of some lipid peroxidation compounds, indicating the individual probability of suffering from early AD.

## Introduction

Alzheimer’s disease (AD) is the main cause of dementia worldwide^1^, and a continuous incidence increase is expected in the next few years with the corresponding great social and economic impact^2^. Nowadays, the standard diagnosis consists of specific neuroimaging procedures and biomarkers in cerebrospinal fluid (CSF)^3,4^, with the corresponding disadvantages of high cost and invasive sampling. In addition, available treatments have proven to be more effective in early stages^5,6^. Therefore, it would be very useful to develop an early and non-invasive screening model based on the individual risk to develop the AD.

In recent years, there is an increase body research about the involvement of oxidative stress since early AD stages^7–9^. Specifically, lipid peroxidation plays an important role in the development of AD due to the high lipid composition of the brain, as well as its high oxygen consumption^10^. In fact, Benseny-Cases *et al*. observed co-localization of oxidized lipids with senile plaques^11^, but also higher levels of oxidized lipids were found in plasma from AD patients than healthy individuals^12^. Among the lipid peroxidation products, isoprostanes (IsoPs) are considered important potential biomarkers of brain damage in AD^13^. Actually, high levels of F_2_-IsoPs were found in CSF^14,15^, as well as in plasma and serum from early AD patients^16,17^. However, few studies have been carried out in urine samples^18–20^ and most of them did not develop any statistical models for predicting AD.

The regression models constitute an important tool to predict the individual risk of suffering from AD. In this sense, few AD predictive models using sophisticated statistical tools can be found in literature, and they are based on CSF biomarkers or not validated analytical methods^21–23^. In addition, most of them required expensive neuroimaging measures^24^. In this study we have developed regression models from lipid peroxidation biomarkers in urine in order to obtain a non-invasive and early AD screening approach.

## Materials and Methods

### Study design and participants

Participants were from the Neurology Unit (University and Polytechnic Hospital La Fe, Valencia, Spain). Their ages were between 50 and 75 years, and they were classified into early AD (case group) (n = 70) and healthy (control group) (n = 29) according to neuropsychological tests, neuroimaging (nuclear magnetic resonance, computerized axial tomography), and CSF biomarkers (β-amyloid, total tau (t-Tau), phosphorylated tau (p-Tau)).

The study protocol was approved by the Ethics Committee (CEIC) from Health Research Institute La Fe (Valencia, Spain), the methods were carried out in accordance with the relevant guidelines and regulations, and informed consent from all participants was obtained.

### Lipid peroxidation compounds

The isoprostanes’ standards 5-F_2t_-IsoP, 2,3-dinor-15-*epi*-15-F_2t_-IsoP, 15(*R*)-15-F_2t_-IsoP, 15-F_2t_-IsoP, 15-E_2t_-IsoP, 15-keto-15-E_2t_-IsoP, 15-keto-15-F_2t_-IsoP, prostaglandins PGE_2,_ PGF_2α_, and 1a,1b-dihomo-PGF_2α_ and deuterated internal standard (IS) PGF_2α_-d_4_ were from Cayman Chemical Company (Ann Arbor, Michigan, USA). The standards of 10-*epi*-10-F_4t_-NeuroP, d_4_-10-*epi*-10-F_4t_-NeuroP, 4(*RS*)-4-F_4t_-NeuroP, 14(*RS*)-14-F_4t_-NeuroP, 7(*RS*)-*ST*-Δ^8^-11-dihomo-IsoF, 17(*RS*)-10-*epi*-SC-Δ^15^-11-dihomo-IsoF, 17-*epi*-17-F_2t_-dihomo-IsoP, 17-F_2t_-dihomo-IsoP, and *ent*-7(*RS*)-7-F_2t_-dihomo-IsoP, were synthesized at the Institute of Biomolecules Max Mousseron (IBMM) (Montpellier, France) by Professor Durand’s team^25^.

The calibration curves were prepared by serial dilutions in H_2_O (pH 3):CH_3_OH (85:15 v/v) with CH_3_COOH 0.01%, in concentrations from 300 nmol L^−1^ to 0.004 nmol L^−1^ of each analyte.

### Urine samples analysis

Urine samples (n = 99) were collected in a sterile bottle and immediately stored at −80 °C until analysis (~6 months). As stated in a previous study, no deterioration was observed for the lipid peroxidation compounds at long-term, since samples were not subjected to freeze-thaw cycles^26^. Then, they were treated following the optimum procedure established in a previous work^26^. Briefly, samples were thawed on ice and 5 μL of the internal standard solution (PI) (PGF_2α_-d_4_ 10 μmol L^−1^ and d_4_-10-*epi*-10-F_4t_-NeuroP 6 μmol L^−1^) were added to 1 mL of sample. Then, enzymatic hydrolysis was performed by adding the enzyme β-glucuronidase and sodium acetate buffer (100 mmol L^−1^, pH 4.9) and incubated for 2 hours at 37 °C. Then, the reaction was stopped and the enzyme was precipitated with cold methanol and chlorhydric acid (37%, v/v) and centrifuged for 10 min (14000 g, 4 °C). The supernatant pH was adjusted to 6–7 with sodium hydroxide (2.5 mol L^−1^). Then, a cleaning and pre-concentration step was carried out by solid-phase extraction (SPE). For this, the cartridges were first conditioned with methanol and H_2_O, then the samples were loaded into the SPE cartridge and the cartridge was washed with ammonium acetate (100 mmol L^−1^, pH 7) and heptane. Elution was carried out with 2 × 500 μL of methanol (5% v/v CH_3_COOH). After that, the samples were evaporated in the vacuum evaporator and reconstituted in 100 μL of H_2_O (pH 3):CH_3_OH (85:15 v/v) containing 0.01% (v/v) CH_3_COOH. Finally, the samples were injected into a chromatographic system (UPLC-MS/MS).

The results were standardized by the creatinine levels measured using a colorimetric kit (MicroVue creatinine EIA) and a spectrophotometer.

### Chromatographic system

The chromatographic system consisted of a UPLC system (Waters Acquity) coupled to a Xevo TQD system mass spectrometry system (Waters, United Kingdom). The conditions used were: ionization in negative mode (ESI-), capillary tension 2.0 kV, source temperature of 150 °C, desolvation temperature of 395 °C, gas flow of the nitrogen cone of 150 L h^−1^, and desolvation flow of 800 L h^−1^.

The LC conditions were selected to achieve appropriate chromatographic retention and resolution by using a C_18_ column (2.1 × 100 mm, 1.7 μm) (Acquity UPLC BEH, Waters). Mobile phases consisted of water (0.01% v/v CH_3_COOH as mobile phase A) and acetonitrile (0.01% v/v acetic acid as mobile phase B). The temperatures of the column and the autosampler were set at 55 °C and 4 °C, respectively. The injection volume was set at 8 µL and the flow rate was set to 0.45 mL min^−1^. A total 8.5 min elution gradient was performed. It consisted of 0.5 min with eluent composition at 80% A and 20% B, which was gradually changed to 55% A and 45% B at 6 min; then B was increased to 95% along 0.2 min, and kept constant for 0.8 min. Finally, the mobile phase composition returned to the initial conditions, and it was maintained for 1.3 min for system conditioning.

The detection was performed by multiple reaction monitoring (MRM) using the acquisition parameters obtained in a previous work^26^.

### Statistical analysis

Data were summarized using median and interquartile range (IQR) in the case of continuous variables, and with relative and absolute frequencies in the case of categorical variables (Table 1). Prior to modelling, variables were log-transformed to avoid potential strongly influential outliers due to the highly skewed nature of some variables (Fig. S1 in Supplementary Material). Then, a logistic regression model based on elastic-net-penalized was developed including gender and age as covariates. The penalization parameter lambda was selected by performing 500 replications of ten-fold cross validation. The minimum cross-validated error was selected on each replication and the median from the selected lambda values was considered the consensus lambda. Since the minimum lambda value was used, an alternative variable selection method was performed as a sensitivity analysis. This alternative analysis consisted on a random forest using the Altmann *et al*. method^27^. The final elastic net model was validated using bootstrap validation. For this, the procedure of Steyerberg *et al*. was followed^28^. Statistical analyses were performed using the softwares R (version 3.5.0), the BootValidation R (version 0.1.3), glmnet R (version 2.0–16), and ranger (version 0.9.0).

**Table 1:** Demographic and clinical variables of the study participants.

Variable	Case (n = 70)	Control (n = 29)
Age (years) (median (IQR))	70.5 (68, 74)	66 (62, 72)
Gender (female) (n (%))	28 (40%)	18 (62%)
Secondary Studies (n (%))	10 (14%)	10 (34%)
Alcohol consumption (yes) (n (%))	6 (8%)	6 (21%)
Smoking status (yes) (n (%))	8 (11%)	1 (3%)
Medications (yes) (n (%))	54 (77%)	18 (62%)
Comorbidity (yes) (n (%))	53 (76%)	18 (62%)
^a^RBANS.DM (median (IQR))	44 (40, 49)	100 (91, 106)
^b^CDR (median (IQR))	0.5 (0.5,1)	0 (0,0)
^c^FAQ (median (IQR))	7 (3, 13)	0 (0, 0)
^d^MMSE (median (IQR))	22 (18, 26)	30 (28, 30)
CSF Amyloid β (pg mL^−1^) (median (IQR))	568 (441, 668)	1227 (1143, 1144)
CSF t-Tau (pg mL^−1^) (median (IQR))	553 (377, 790)	208 (141, 333)
CSF p-Tau (pg mL^−1^) (median (IQR))	88 (71, 116)	51 (38, 70)
Temporal atrophy (yes) (n (%))	51 (72%)	2 (7%)
Depression (yes) (n (%))	9 (13%)	3 (10%)

IQR: Interquartilic range.

^a^RBANS-DM, Repeatable Battery for the Assessment of Neuropsychological Status- Delayed Memory (Standard Score; cut-off point < 85).

^b^CDR, Clinical Dementia Rating, values: 0, 0.5, 1, 2.

^c^FAQ, Functional Activities Questionnaire (Direct Score; cut-off point >9).

^d^MMSE, Minimental State Examination.

## Results

### Participants’ characteristics

Table 1 shows the demographic and clinical data for both groups. Small differences were shown for age and gender between groups, so these variables were considered covariates. Regarding the neuropsychological variables (Clinical Dementia Rating (CDR), Repeatable Battery for the Assessment of Neuropsychological Status (RBANS), Functional Activities Questionnaire (FAQ), Minimental State Examination (MMSE) and biological measures (CSF β-amyloid, CSF t-Tau, CSF p-Tau, temporal atrophy) used in the standard diagnosis, they showed significant differences between groups. However, the demographic variables (age, gender, studies, alcohol, smoking status, medication and comorbidity) did not show statistical differences between groups.

### Determination of urine lipid peroxidation biomarkers

**Figure 1:**
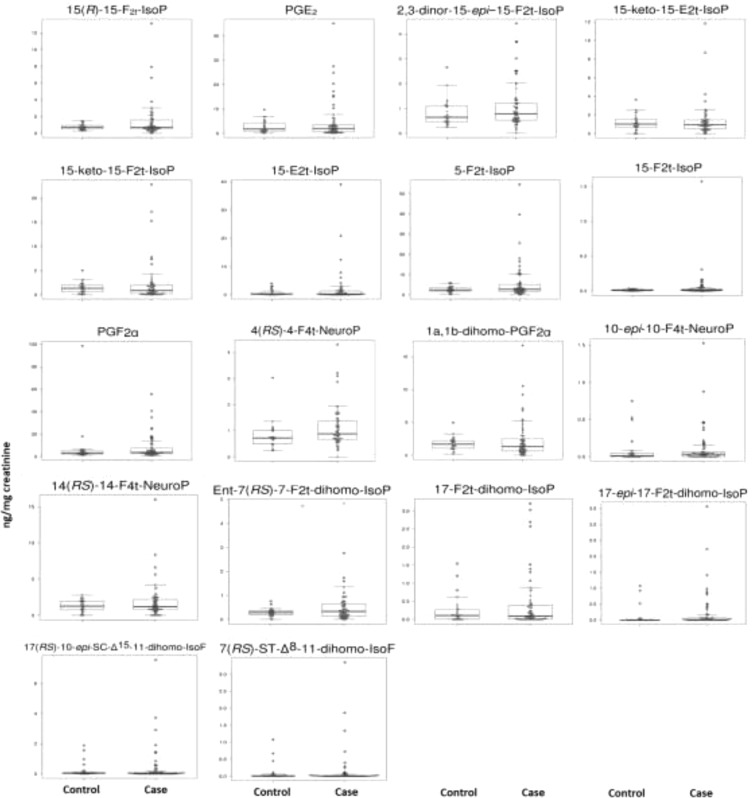
Box-Plot of the differences in different lipid peroxidation analytes levels between early AD (case) and healthy (control) groups.

Urine levels of lipid peroxidation compounds obtained for each group are shown in Table 2. Some of them (5-F_2t_-IsoP, 2,3-dinor-15-epi-15-F_2t_-IsoP, 15-E_2t_-IsoP, PGE_2_, PGF_2α_, 10-*epi*-10-F_4t_-NeuroP, 4(*RS*)-4-F_4t_-NeuroP, *ent*-7(*RS*)-7-F_2t_-dihomo-IsoP) showed higher levels in early AD patients than in healthy controls, and some analytes (15-keto-15-E_2t_-IsoP, 15-keto-15-F_2t_-IsoP) showed lower values in the case group than in the control group. Figure 1 shows the box plots for each analyte.

**Table 2:** Concentrations of lipid peroxidation biomarkers in urine samples.

Biomarkers	Case (n = 70)	Control (n = 29)
	Median (IQR) (ng mg^−1^ creatinine)	Median (IQR) (ng mg^−1^ creatinine)
15(*R*)-15-F_2t_-IsoP	0.72 (0.5, 1.56)	0.7 (0.48, 0.94)
PGE_2_	1.98 (0.62, 3.5)	1.69 (0.93, 4.26)
15-keto-15-E_2t_-IsoP	0.93 (0.53, 1.47)	1.02 (0.65, 1.54)
15-keto-15-F_2t_-IsoP	0.84 (0.22, 1.94)	1.33 (0.58, 2)
2,3-dinor-15-epi-15-F_2t_-IsoP	0.78 (0.53, 1.22)	0.65 (0.47, 1.09)
15-E_2t_-IsoP	0.23 (0.06, 1.31)	0.16 (0.07, 0.58)
5-F_2t_-IsoP	2.67 (1.68, 5.07)	2.37 (1.76, 3.37)
15-F_2t_-IsoP	0.01 (0, 0.02)	0.01 (0, 0.02)
PGF_2α_	3.72 (2.79, 7.32)	3.38 (2.35, 5.17)
4(*RS*)-4-F_4t_-NeuroP	0.89 (0.67, 1.36)	0.72 (0.5, 1.01)
1a,1b-dihomo-PGF_2α_	1.33 (0.64, 2.48)	1.67 (1.05, 2.23)
10-*epi*-10-F_4t_-NeuroP	0.03 (0, 0.06)	0.01 (0, 0.05)
14(*RS*)-14-F_4t_-NeuroP	1.21 (0.76, 2.16)	1.27 (0.74, 1.94)
*ent*-7(*RS*)-7-F_2t_-dihomo-IsoP	0.33 (0.14, 0.63)	0.28 (0.19, 0.36)
17-F_2t_-dihomo-IsoP	0.09 (0, 0.38)	0.11 (0, 0.26)
17-*epi*-17-F_2t_-dihomo-IsoP	0.01 (0, 0.07)	0 (0, 0)
17(*RS*)-10-*epi*-SC-Δ^15^-11-dihomo-IsoF	0.03 (0, 0.1)	0.05 (0.03, 0.08)
7(*RS*)-ST-Δ^8^-11-dihomo-IsoF	0 (0, 0.02)	0 (0, 0.03)

IQR, inter-quartile range; IsoP, isoprostane; dihomo-IsoP, dihomo-isoprostane; dihomo-IsoF, dihomo-isofuran, NeuroP, neuroprostane;

### Screening model from urine lipid peroxidation biomarkers

The elastic net model selected five variables corresponding to one isoprostane, one neuroprostane, one prostaglandin and two dihomo-isoprostanes shown in Table 3. The model also included gender and age, which were introduced as covariates. These predictor variables were combined as it is indicated in the formula below in order to estimate the individual probability (Pr) of suffering from AD.

$$\Pr (Y)\frac{{e}^{-4.187+0.463\ast female+0.064\ast age-0.13\ast (A)+0.622\ast (B)-0.048\ast (C)+0.554\ast (D)+0.072\ast (E)}}{1+{e}^{-4.187+0.463\ast female+0.064\ast age-0.13\ast (A)+0.622\ast (B)-0.048\ast (C)+0.554\ast (D)+0.072\ast (E)}}$$

**Table 3:** Results of the elastic net and random forest analyses.

Variable	Coefficient (elastic net)	Importance (random forest)	p-value (random forest)
Gender (female)	0.463	0.17	0.08*
Age	0.064	1.09	0.012*
15-keto-15-F_2t_-IsoP	−0.13	0.71	0.043*
4(*RS*)-F_4t_-NeuroP	0.62	0.74	0.046*
1a,1b-dihomo-PGF_2α_	−0.048	0.73	0.035*
*ent*-7(*RS*)-7-F_2t_-dihomo-IsoP	0.55	0.64	0.044*
17-*epi*-17-F_2t_-dihomo-IsoP	0.072	0.58	0.029*
10-*epi*-10-F_4t_-NeuroP	0	0.48	0.075
17-F_2t_-dihomo-IsoP	0	0.35	0.133
17(*RS*)-10-*epi*-SC-Δ^15^-11-dihomo-IsoF	0	0.21	0.219
15-E_2t_-IsoP	0	0.17	0.293
5-F_2t_-IsoP	0	0.14	0.325
2,3-dinor-15-*epi*-15-F_2t_-IsoP	0	0.11	0.381
15(*R*)-15-F_2t_-IsoP	0	0.10	0.379
PGE_2_	0	0.08	0.405
15-keto-15-E_2t_-IsoP	0	0.05	0.436
7(*RS*)-ST-Δ^8^-11-dihomo-IsoF	0	−0.08	0.636
PGF_2α_	0	−0.09	0.603
14(*RS*)-14-F_4t_-NeuroP	0	−0.25	0.755

Coefficients of the elastic net model are interpreted as log-odds, so negative values indicate a negative association between higher concentration levels and risk of disease and positive values indicate a positive association between higher concentration levels and risk of disease. Importance values and p-values for random forest are derived from the gini index using Altman method.

A: 15-keto-15-F_2t_-IsoP; B: 4(*RS*)-4-F_4t_-NeuroP; C: 1a,1b-dihomo-PGF_2α_; D: *ent*-7(*RS*)-7-F_2t_-dihomo-IsoP; E: 17-*epi*-17-F_2t_-dihomo-IsoP

**Figure 2:**
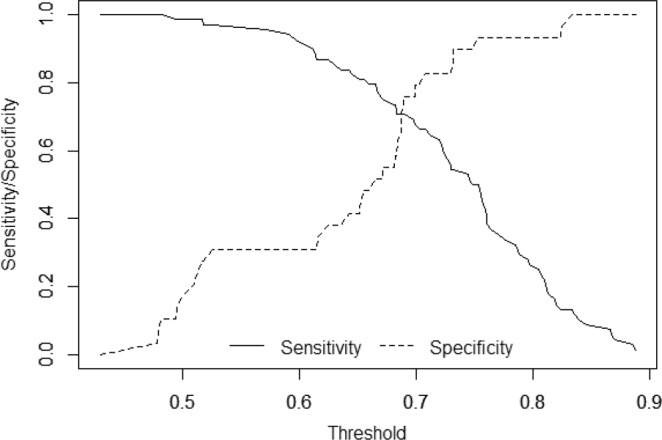
Sensitivity and specificity profile plot. The continuous line depicts the relationship between the probability threshold set in the model’s prediction and its corresponding sensitivity and the dashed line represent the relationship between the probability threshold and the specificity.

The alternative analysis using random forest selected the same five variables as the most important ones (Table 3), and they were also all considered statistically significant by the Altmann method^27^. Classification performance of the models was assessed using bootstrap in the case of elastic net and by the Out of Bag (OOB) estimate in the case of random forest. Bootstrap validated area under the receiver operating characteristics (AUC-ROC) for the elastic net model was 0.682 and OOB accuracy for the random forest model was 0.71, so their performance can be considered similar. Remarkably for the elastic net results, the sensitivity and specificity profile shows a sharp decrease of the sensitivity values as the specificity increases, forcing a decision between high sensitivity (0.97) at a cost of low specificity (0.31) or high specificity (0.93) at a cost of mediocre sensitivity (0.5) (Fig. 2).

## Discussion

The reliable determination of lipid peroxidation products levels in urine samples from well-defined healthy and early AD participants, and the satisfactory classification performance of two complementary regression models allowed to develop an early and non-invasive screening model to identify individuals with high risk to develop the AD.

The role of lipid peroxidation in AD development has been largely studied^10^, but few studies have been carried out determining isoprostanoids as target metabolites in AD^17,29^. In addition, the analytical methods used in most of these works were based on commercial kits or immunoassays what is associated to low specificity on isomers determinations^30^. Nevertheless, in the present study a previously validated analytical method based on mass spectrometry detection has been used, providing high selectivity and sensitivity, as well as high reliability to determine simultaneously several isoprostanoids isomers^26^.

Regarding the development of early and non-invasive diagnosis, urine could be considered a promising matrix. However, few studies in literature have focused on this matrix^31,32^. Specifically, in the present work some compounds (PGE_2_, 2,3-dinor-15-epi-15-F_2t_-IsoP, 15-E_2t_-IsoP, 5-F_2t_-IsoP, PGF_2α_, 10-*epi*-10-F_4t_-NeuroP, 4(*RS*)-4-F_4t_-NeuroP and 17-*epi*-17-F_2t_-dihomo-IsoP) showed higher concentrations in urine from AD patients than in healthy participants. Similarly, previous studies showed higher levels of some F_2_-IsoPs in urine from patients with AD than in the control group^18–20^. However, further studies to clinically validate these potential biomarkers, using a larger number of samples from well-defined participants, and predictive models are required.

In this work, two alternative modeling methods with completely different characteristics were used. First, elastic net logistic regression is based on standard generalized linear regression models, thus assuming linearity of the relationship between predictors and the linear predictor, no interactions are assessed and the results are fully interpretable as in a standard logistic regression. On the other hand, random forest is a non-linear non-parametric model, that enable the assessment of higher order interactions between variables at a cost of lower statistical power compared to elastic net model when the relationship is linear^33,34^. Random forest does not provide an interpretable model, but provides a list of the most important variables in predicting the response. The fact that both methods obtained very similar results, provides robustness to our results.

In literature, few AD predictive models using these sophisticated statistical tools can be found^21–23,34^, and most of them are based on neuroimaging measures^24^. However, none of them were based on non-invasive determination of lipid peroxidation biomarkers in early AD patients.

The diagnostic indexes obtained from both models indicated that the results could constitute a satisfactory screening approach from early AD stages with the consequent benefits for patients and health public system. In fact, the high sensitivity obtained would allow a reliable identification of high-risk patients in the early stages of AD, and they would be derived to a method with higher specificity to rule out false positives^17^. Nevertheless, further clinical validation using an external cohort of participants would be required in order to obtain a reliable diagnostic model.

Regarding the study limitations, the low number of controls compared to cases would be explained by the difficulty to obtain healthy participants with CSF biomarkers. Also, we did not include participants with other similar dementias, so differential AD diagnosis was not achieved. Further clinical validation work will be developed by including a higher number of controls, as well as patients with similar pathologies. In addition, a follow-up study will be carried out in order to evaluate the variation of these compounds levels along the time.

## Conclusion

A set of new lipid peroxidation biomarkers has been determined in urine samples from well-defined participants (early AD, healthy) by means of a previously validated analytical method. So, reliable results have been obtained and used to develop a preliminary early and non-invasive screening model in order to identify potential individuals with high risk of suffering AD, although it could not be considered AD specific. For this, two different regression models (linear, elastic net; non-linear, random forest) were developed, obtaining similar performance in terms of variable selection and accuracy, in spite of being based on different analytical principles, and so providing robustness to the results.

## Supplementary information

Figure S1. Distribution of the log-transformed data for all the variables

## Data Availability

The datasets generated during the current study are available from the corresponding author on reasonable request.
