# Supplementary material for: New screening approach for Alzheimer’s disease risk assessment from urine lipid peroxidation compounds
Source: Sci Rep. 2019 Oct 2;9:14244. doi: 10.1038/s41598-019-50837-2 (PMC6775072; doi:10.1038/s41598-019-50837-2)
Supplement: Supplementary file 1 — Figure S1. Distribution of the log-transformed data for all the variables [file 41598_2019_50837_MOESM1_ESM.pdf]

New screening approach for Alzheimer's disease risk assessment from urine lipid peroxidation compounds

Carmen PEÑA-BAUTISTA 1, Claire VIGOR 2, Jean-Marie GALANO 2, Camille OGER 2, Thierry DURAND 2, Inés FERRER 3, Ana CUEVAS 3, Rogelio LÓPEZ-CUEVAS 3, Miguel BAQUERO 3, Marina LÓPEZ-NOGUEROLES 4, Máximo VENTO 1, David HERVÁS-MARÍN 5, Ana GARCÍA-BLANCO 1,*, Consuelo CHÁFER-PERICÁS1,*

1Neonatal Research Unit, Health Research Institute La Fe, Valencia, Spain
2Institut des Biomolécules Max Mousseron, IBMM, University of Montpellier, CNRS ENSCM, Montpellier, France
3Neurology Unit, University and Polytechnic Hospital La Fe, Valencia, Spain
4Analytical Unit Platform, Health Research Institute La Fe, Valencia, Spain
5Biostatistical Unit, Health Research Institute La Fe, Valencia, Spain

Corresponding author*
Consuelo Cháfer-Pericás, PhD
Health Research Institute La Fe
Avda de Fernando Abril Martorell, 106; 46026 Valencia (Spain)
Phone: +34 96 124 66 61, Fax: + 34 96 124 57 46
Email address: m.consuelo.chafer@uv.es
Ana.garcia-blanco@uv.es

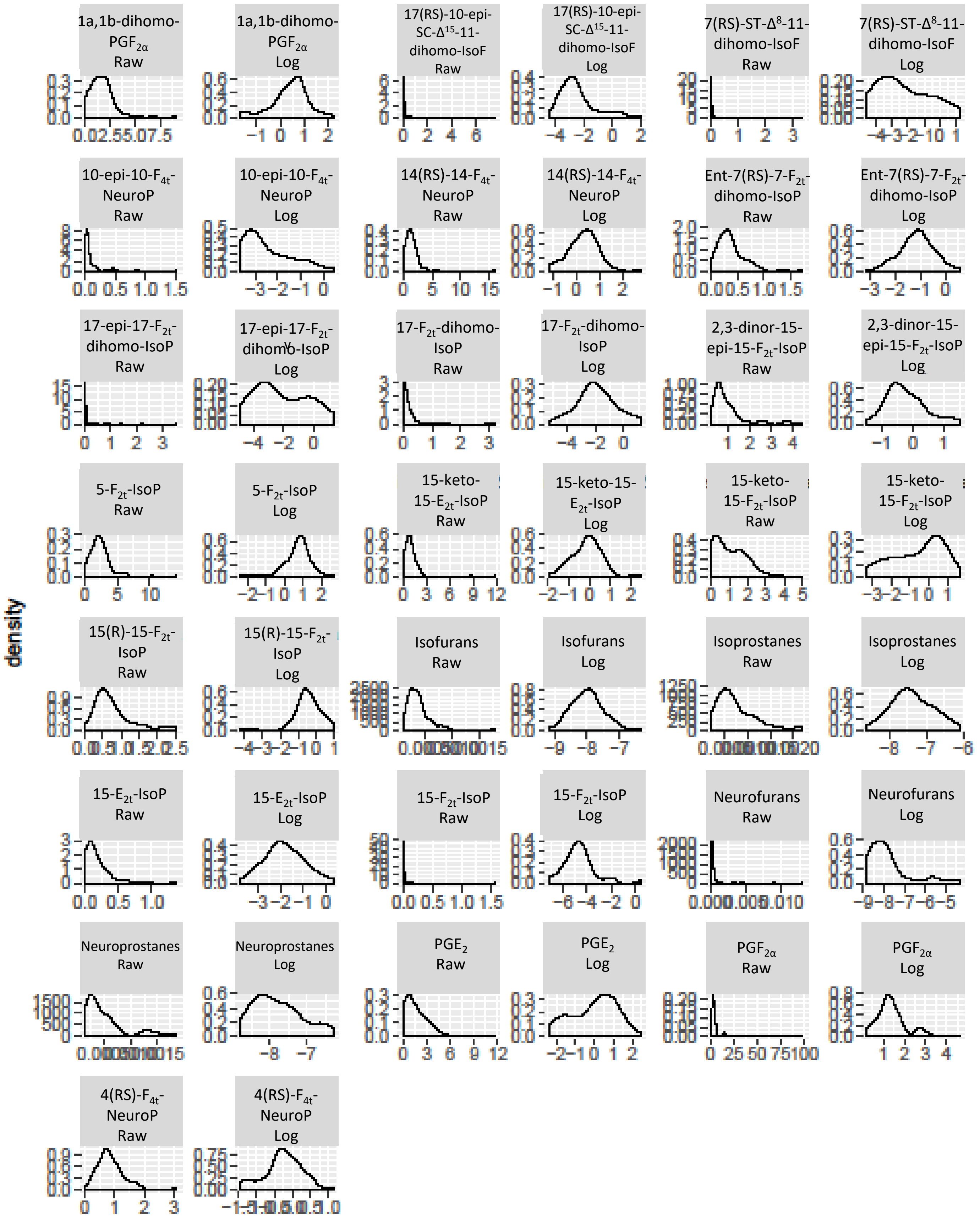

Supplementary Material

Figure S1. Distribution of the log-transformed data for all the variables.
